# Azimuthal rotation-controlled nanoinscribing for continuous patterning of period- and shape-tunable asymmetric nanogratings

**DOI:** 10.1038/s41378-024-00687-4

**Published:** 2024-05-11

**Authors:** Useung Lee, Hyein Kim, Dong Kyo Oh, Nayeong Lee, Jonggab Park, Jaewon Park, Hyunji Son, Hyunchan Noh, Junsuk Rho, Jong G. Ok

**Affiliations:** 1 Department of Mechanical and Automotive Engineering, Seoul National University of Science and Technology, Seoul, 01811 Republic of Korea; 2 Department of Mechanical Engineering, Pohang University of Science and Technology (POSTECH), Pohang, 37673 Republic of Korea; 3 Department of Chemical Engineering, Pohang University of Science and Technology (POSTECH), Pohang, 37673 Republic of Korea; 4 POSCO-POSTECH-RIST Convergence Research Center for Flat Optics and Metaphotonics, Pohang, 37673 Republic of Korea; 5 National Institute of Nanomaterials Technology (NINT), Pohang, 37673 Republic of Korea; 6 Present Address: Department of Mechanical Engineering, Korea University, 145 Anam-ro, Seongbuk-gu, Seoul, 02841 Republic of Korea; 7 Present Address: Research Team, Hyundai Motor Group, 150 Hyundaiyeonguso-ro, Hwaseong-si, Gyeonggi 18280 Republic of Korea

**Keywords:** Optical materials and structures, Nanofabrication and nanopatterning

## Abstract

We present an azimuthal-rotation-controlled dynamic nanoinscribing (ARC-DNI) process for continuous and scalable fabrication of asymmetric nanograting structures with tunable periods and shape profiles. A sliced edge of a nanograting mold, which typically has a rectangular grating profile, slides over a polymeric substrate to induce its burr-free plastic deformation into a linear nanopattern. During this continuous nanoinscribing process, the “azimuthal angle,” that is, the angle between the moving direction of the polymeric substrate and the mold’s grating line orientation, can be controlled to tailor the period, geometrical shape, and profile of the inscribed nanopatterns. By modulating the azimuthal angle, along with other important ARC-DNI parameters such as temperature, force, and inscribing speed, we demonstrate that the mold-opening profile and temperature- and time-dependent viscoelastic polymer reflow can be controlled to fabricate asymmetric, blazed, and slanted nanogratings that have diverse geometrical profiles such as trapezoidal, triangular, and parallelogrammatic. Finally, period- and profile-tunable ARC-DNI can be utilized for the practical fabrication of diverse optical devices, as is exemplified by asymmetric diffractive optical elements in this study.

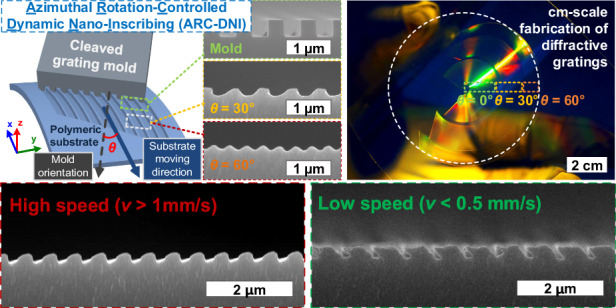

## Introduction

Asymmetric nanogratings, such as slanted or blazed nanogratings exhibit high diffraction efficiency, which helps to improve the sensitivity, resolution, and measurement range of grating-based displacement measurement systems. Thus, these slanted and blazed nanogratings are useful in many optical applications, such as optical instruments (e.g., spectrometers and monochromators)^1–3^, miniaturized sensors^4^, head-up displays^5,6^, and diverse active optical devices^7,8^. In particular, diffractive optical elements (DOEs), which control the wavefront of electromagnetic waves, have emerged because they can be combined with other optical components and constituted in a tiny volume^9^. For instance, a DOE in conjunction with liquid crystal layers can increase first-order diffraction efficiency from 60% to 90%^10^. By employing asymmetric or slanted nanogratings, DOEs with higher diffraction efficiency can be used to modulate various properties of light, such as its spatial distribution, phase, and polarization^11,12^, which makes them promising for use in virtual/augmented reality (VR/AR) devices and high-resolution imaging systems^13^.

However, reliable and cost-effective fabrication of asymmetric nanograting patterns with well-controlled slanted/blazed profiles and specific periods remains challenging. Conventional lithography techniques (*e.g*., photolithography, electron-beam lithography, laser interference lithography) often require complex and expensive procedures that involve multiple pattern-mask preparation steps and delicate oblique etching processes^14–16^. Direct machining approaches, such as imprinting^17–21^, mechanical ruling^22^, focused ion-beam milling^23^, and direct laser writing^24,25^, are alternative approaches for fabricating asymmetric/slanted gratings with variable depths for use in flexible devices^26–28^. While these methods are more effective than lithography, they are not scalable because they still need larger-area mold or masks, longer processing time, or more expense to practically fabricate optical devices^29–31^. Likewise, DOEs can be practically fabricated by adopting new lithographic materials^32^ or a mechanical patterning process^33^, but they still have symmetric grating shapes that are limited to conventional functions of DOEs. For these reasons, methods that can be used to create asymmetric nanograting patterns with tailored periods, shapes, and slopes at a high speed over a large area, without using large-area molds or masks and complex apparatuses, are needed urgently.

Recent studies have demonstrated that nanograting patterns with a specific period (pitch) and depth can be inscribed mechanically by linearly sliding one edge of a rigid nanograting “tool” on a viscoelastic polymer surface with a controlled force, temperature, and speed^34–38^. In such a case, the tool can be prepared readily by cleaving a Si or SiO_2_ wafer (typically along the (100)-plane) containing the desired nanograting pattern across grating lines, where the original nanograting pattern on the wafer piece tool typically has a symmetric (*i.e*., rectangular or isosceles trapezoidal) profile^35–37^. This continuous nanoinscribing technique facilitates scalable and high-speed fabrication of nanograting patterns on flexible polymers by using a simple process. Also, this nanoinscribing process leaves no burr and residue behind because the inscribing tool simply pushes away the pattering region in the polymeric substrate, unlike the conventional mechanical milling process. Moreover, the rigid tool is hardly damaged by soft polymers, thus the patterning area is ~100 times larger than the original tool area, securing high scalability and repeatability^35^. However, the pitch of the inscribed nanograting is governed by the original pitch of the tool, and the profile of the inscribed nanograting is limited to symmetrical shapes because of the original symmetric profile of the tool. The linear inscription stroke of a nanograting-bearing tool along the orientation parallel to its grating lines will likely be unable to create asymmetric and slanted nanograting patterns owing to the original symmetric pattern profile in such cases^35,38^.

**Fig. 1:**
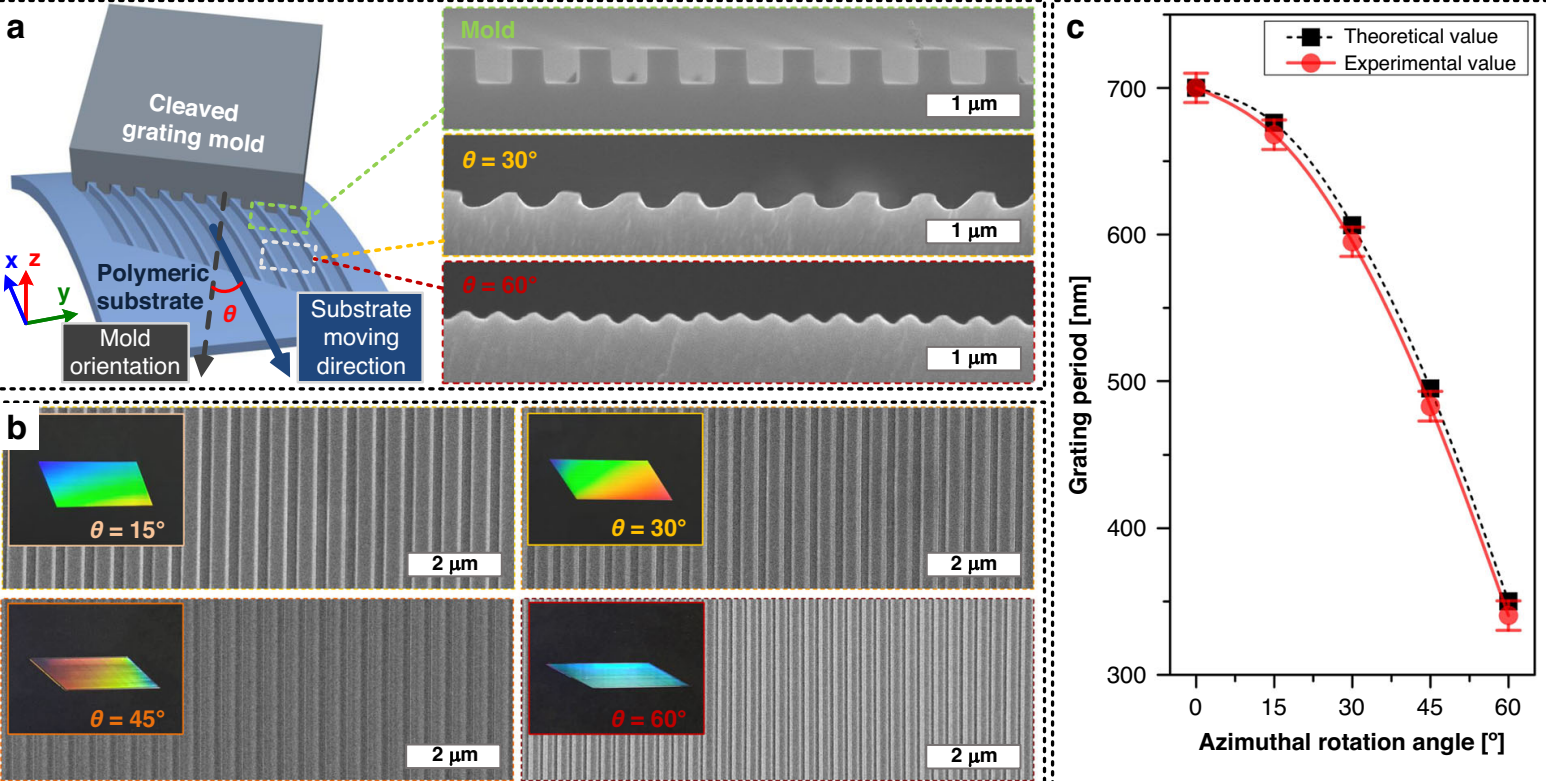
Azimuthal rotation-controlled dynamic nanoinscribing (ARC-DNI): process principle and tunability of nanograting period. **a** Scheme of azimuthal rotation-controlled dynamic nanoinscribing (ARC-DNI) process and cross-sectional SEM images of mold and representative asymmetric nanogratings fabricated by controlling azimuthal angle (*θ*) during ARC-DNI. *θ* is the angle between the mold grating line and the inscription stroke line, and it can be controlled by rotating the mold along the z-axis, as indicated in the lower-left corner. **b** Optical and top-view SEM images of the nanogratings fabricated using ARC-DNI at *θ* values of 15°, 30°, 45°, and 60° on PC substrates at the temperature, force, and inscribing speed of 150 °C, 2 N, and 1 mm/s, respectively. **c** Calculated and measured periods of the nanogratings fabricated using ARC-DNI at various *θ* values

To address the challenges described above, in this work, we propose a method for scalable, continuous, and high-speed fabrication of period- and shape-tunable asymmetric nanograting patterns termed “azimuthal rotation-controlled dynamic nanoinscribing (ARC-DNI).” Here, the azimuthal angle represents the angle between the direction of the inscription stroke and the nanograting line orientation of the tool (Fig. 1a). First, we show that the period of the inscribed nanograting pattern can be tuned readily by controlling the azimuthal angle of a single patterning tool, a process that cannot be performed readily when using the existing nanopatterning methods, including the aforementioned typical inscription process. More importantly, we demonstrate that ARC-DNI allows for the facile fabrication of asymmetric nanograting patterns with various geometrical profiles, including nonisosceles trapezoids, parallelograms, and triangles, by controlling polymeric reflow at different azimuthal angles. Interestingly, it becomes possible to tailor nanograting shapes over such a wide range by controlling not only the azimuthal angle but also other process parameters, including temperature, contact force, and stroke speed of the inscribing tool (referred to as the “mold” hereinafter). The effects of these parameters are analyzed systematically and discussed in this paper. Among the many possible applications of ARC-DNI, as an example, we describe the fabrication of a diffractive grating with asymmetric slanted profiles by applying ARC-DNI at various azimuthal angles.

## Results and Discussion

### Continuous fabrication of period-tunable nanogratings by controlling azimuthal angle in ARC-DNI

Figure 1 shows how ARC-DNI can be used for continuous patterning of nanograting structures with various periods by controlling the azimuthal angle (*θ*; angle along the *z*-axis, as indicated in Fig. 1a and Fig. S1). The three-dimensional (3D) scheme and representative aspects of ARC-DNI are depicted in Fig. 1a. A well-cleaved rigid mold with an edge exhibiting typical rectangular nanopattern slides over a flexible substrate at a specific *θ* continuously to create nanogratings having diverse periods and profiles (*θ* = 30° and 60° cases are presented as examples). For clearer definition and understanding of *θ*, the normal x-y plane view and the simplified spherical coordinate view of the ARC-DNI process system are described in Fig. S1 in the *Supplementary Information*. Here, the mold and substrate are in conformal contact under a controlled contact force and temperature and *θ* is also precisely controlled by fixing the substrate rotation angle. Additional images of the three-dimensional CAD model and prototype of the ARC-DNI system with the graphical definition of *θ* are presented in Fig. S2, and the detailed experimental procedure is presented in the *Experimental and Methods* section. In this section, we focus on facile tuning of nanograting periods by simply controlling *θ* values. Specifically, the period (*λ*) of a nanograting fabricated using ARC-DNI can be expressed as follows:

1 $$\lambda ={\lambda }_{{mold}}\times \cos (\theta )$$

where *λ*_*mold*_ is the period (pitch) of the original nanopattern in the mold. Figure 1b depicts scanning electron microscopy (SEM) images and optical photos of four different nanograting structures fabricated on PC films (unless specified otherwise) by using one identical nanopattern mold with a period of 700 nm at different *θ* values of 15°, 30°, 45°, and 60°; the processing temperature, force, and inscribing speed are 150 °C, 2 N, and 1 mm/s, respectively. The nanograting periods fabricated in each case precisely match those calculated using Eq. (1), as shown in Fig. 1c. Notably, the data points are obtained by averaging the values measured in the same conditions for three to five times, which indicates that the ARC-DNI process is reproducible for the fabrication of nanogratings with specific periods. The reflected colors of the samples change as the periods (and shapes) of the inscribed nanogratings change, as will be demonstrated further by realizing diffractive blazed gratings later in this paper.

### Tailoring of asymmetric nanograting profiles through the azimuthal angle-dependent mold-opening profile

**Fig. 2:**
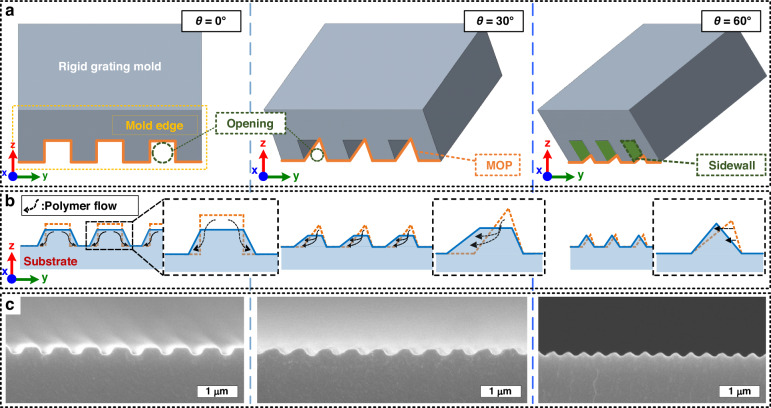
Shaping of asymmetric nanograting profile through the azimuthal angle-controlled mold-opening profile (MOP). **a** Schematic front-view drawings of MOPs (orange lines) corresponding to *θ* values of 0°, 30°, and 60°. **b** Cross-sectional schemes of nanogratings whose final shapes (blue lines) are determined by the MOP-guided as-deformed profiles (orange lines) followed by polymer reflows (black arrows) during ARC-DNI at the corresponding *θ* values for viscoelastic recovery. **c** Cross-sectional SEM images of nanogratings fabricated using ARC-DNI at the corresponding *θ* values. The process temperature, force, and speed were set to 150 °C, 2 N, and 1 mm/s, respectively, in all cases

In the ARC-DNI process, the shape and period of nanograting structures can be controlled by changing *θ* values of the mold. Considering that nanoinscribing is analogous to the conventional extrusion process in which the substrate (workpiece) material is extruded through openings in a mold^35^, the contours of nanogratings fabricated using ARC-DNI can be outlined on the basis of the mold-opening profile (MOP), which changes according to *θ* values (Fig. 2a). During the ARC-DNI process, the substrate material (typically polymeric) undergoes “burr-free” plastic deformation, followed by the viscous flow and elastic recovery based on the temperature-dependent viscoelastic properties of the substrate material^35,38^. In view of these points, we attempt to establish that the final nanograting shape can be determined from the MOP-directed outline and flow characteristics of the polymer material when the values of *θ* and temperature are controlled. Figure 2a depicts the MOPs obtained at *θ* = 0°, 30°, and 60°. At *θ* = 0°, the MOP has a rectangular shape that is identical to the original pattern profile of the mold (i.e., a period of 700 nm (hill-to-valley ratio of 1:1), height of 400 nm, and tilt angle of 35°). When *θ* = 30°, the internal sidewall of the mold’s nanograting (simply called the “sidewall” hereinafter) “eclipses” the opening, thereby shaping the MOP into a scalene triangle. At *θ* = 60°, the opening area decreases further as it is eclipsed to a greater extent by the sidewall, and the MOP is reduced to a small, blazed shape. Practically, the ability to change the MOP simply by controlling *θ* can mitigate the need to prepare multiple molds with different pattern profiles.

Figure 2b illustrates the polymer flows during the ARC-DNI process executed using *θ* values of 0°, 30°, and 60°, and Fig. 2c shows the corresponding actual fabrication results. At *θ* = 0°, the polymer material is inscribed in a rectangular shape through concurrent contact with both the left and right sidewalls, and the final symmetric trapezoidal nanograting is obtained through symmetric bidirectional polymer reflow for viscoelastic recovery^39,40^. At *θ* > 0°, this polymer reflow is directional because the polymer material has different contact times with each sidewall; specifically in the front-view image of the *θ* = 30° case shown in Fig. 2a (and the top-view scheme shown in Fig. 4b), the polymer structure is released from the left sidewall first while still maintaining contact with the right sidewall. This extends the time available for the polymer to unidirectionally reflow toward the left compared to that to reflow toward the right, which, consequently, generates an asymmetric nanograting structure. Notably, the difference between the contact times with each of the sidewalls can be adjusted by controlling the inscribing speed in ARC-DNI, which can facilitate additional tuning of the resulting nanograting shapes. This time-dependent polymer flow dynamics and the corresponding structural tuning results (e.g., parallelogram-like slanted nanograting) are discussed later in this paper (Fig. 4). When *θ* increases further to leave an extremely small triangular opening area, as in the *θ* = 60° case, on the other hand, initial polymer deformation is rather limited by the small MOP, and the polymer reflow decreases accordingly. This limited polymer deformation eventually makes a condensed blazed nanograting structure.

### Tuning depth and shape of asymmetric nanograting by controlling temperature and force during ARC-DNI

**Fig. 3:**
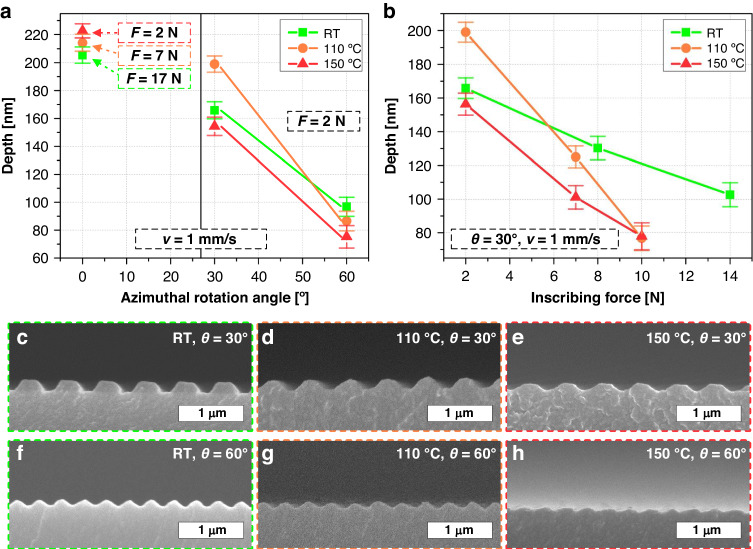
Tailoring of depth and shape of asymmetric nanograting by controlling temperature and force during ARC-DNI. **a**, **b** Depths of nanogratings fabricated by controlling *θ*, *T*, and *F* in ARC-DNI (inscribing speed (*v*) is set to 1 mm/s in all cases). **c**–**h** Cross-sectional SEM images of nanogratings fabricated by ARC-DNI, with the corresponding *T*-*θ* conditions marked in the lower-left corners; in all cases, *F* = 2 N and *v* = 1 mm/s

The ARC-DNI processing temperature (*T*), in conjunction with the inscribing force (*F*) and *θ*, plays a significant role in controlling the resultant nanograting depth and shape by modulating the plastic deformation characteristics of the polymer material. Here, *T* denotes the local temperature of the mold edge, which can be adjusted using the microheater embedded in the mold-attached arm (see the *Experimental and Methods* section for details of this setup). In general, a higher *T* can reduce the viscosity of a polymer^41^ and enhance the malleability of a polymer substrate film within its glass-transition temperature (*T*_*g*_) range, thereby inducing greater deformation under the identical mechanical force provided by the mold’s inscribing stroke. By contrast, at a lower *T*, a larger force would be required to obtain a nanograting with a similar depth and shape. Figure 3 overall shows the *T*-dependent ARC-DNI results at various *F* and *θ* values. To conduct a systematic investigation, we use three values of *T* as follows: 25 °C (room temperature), 110 °C, and 150 °C (for the PC substrate material whose *T*_*g*_ is slightly higher than 150 °C in this case).

We first comment on the *θ* = 0° case, in which symmetric nanogratings are built up through the largest mold-opening area defined by the mold’s original rectangular MOP, as plotted in the left side of Fig. 3a. At *T* = 150 °C, which approaches *T*_*g*_, the nanograting depth can reach 220–225 nm under a relatively small *F* of 2 N. However, the *F* required to obtain nanogratings of similar depths increases from 2 N through 7 N to 17 N as *T* decreases from 150 °C through 110 °C to 25 °C, respectively. This corroborates the aforementioned polymer behavior determined from the *T–F* correlation^41^. The complete *T–F* matrix of fabricated nanogratings by the general DNI process (*θ* = 0°) is presented with SEM images in Fig. S3. As *θ* increases from 0° through 30° to 60°, the MOP-defined mold-opening area decreases, which indicates that the total quantity of polymer material deformed by the mold-inscription-driven mechanical force decreases, as discussed above. This can reduce the *F* required for polymer deformation, while the effect of the *T*-driven, thermally facilitated polymer reflow (simply referred to as “thermal reflow” hereinafter) becomes more pronounced. As *θ* and *T* increase, the margins of the nanograting depth decrease, even at the same relatively small *F* value of 2 N, as indicated on the right side of Fig. 3a. The overall decrease in the nanograting depth as *θ* increases can be attributed mainly to the incremental sidewall eclipsing that reduces the MOP height proportionally, as illustrated in Fig. 2a. Moreover, the depths achievable using ARC-DNI at different temperatures are different because the MOP decreases as *θ* increases and also because the polymeric reflow increases as *T* increases. For instance, at *θ* = 30°, the maximum height is achieved by ARC-DNI at *T* = 110 °C, while at *θ* = 60°, the maximum height is achieved by ARC-DNI at 25 °C. This result is appreciably reasonable in the sense that the MOP decides the initial deformable volume of polymer materials and *T* decides the reflowing degree of deformed polymers, also supported by our previous research that initially deformed volume is subject to *F* and polymeric reflow is controlled by *T*^35^.

We now focus on the *θ* = 30° case. Figure 3b shows plots of the depths of the nanograting structures obtained from the 30° azimuthally rotated ARC-DNI process performed at various *T* and *F* values; the corresponding SEM images are shown in Fig. 3c–e. The first finding is that the nanograting depth decreases as *F* increases for all values of *T*. This can be understood by considering the *F*-dependent changes to the mold-opening height; as *F* increases, the mold’s nanopatterned edge is buried in the flexible polymer substrate with an increased penetration depth. This, in turn, decreases the mold-opening height, leading to a decrease in the nanograting depth achieved by ARC-DNI. More importantly, the nanograting depth decreases (Fig. 3b) and width increases (Fig. 3c–e) as *T* increases. This occurs because the thermal reflow (along the vertical and horizontal directions) of the as-deformed polymer structure becomes more active because of the decrease in viscosity at higher *T* values^41^. The complete *T–F* matrix of the ARC-DNI results in the *θ* = 30° is presented with SEM images in Fig. S4.

In the *θ* = 60° case, ARC-DNI creates blazed nanogratings throughout the *T* range, as depicted in Fig. 3f–h. The MOP-defined mold opening, which is reduced considerably to a very small triangular area, facilitates the faithful formation of blazed nanogratings with similar depths by readily filling the small mold-opening area, regardless of changes in *T* or *F* (the rightmost side of Fig. 3a). Nonetheless, upon a closer examination of Fig. 3b, we find that as *T* increases, the overall nanograting depth decreases with a smaller angular profile (Fig. 3f–h), which is consistent with the above discussion on how a higher *T* facilitates reflow of the as-deformed polymer material by lowering its viscosity. The complete *T–F* matrix of the experimental results obtained by the ARC-DNI process in the *θ* = 60° case is presented in Fig. S5, which clearly shows the *T*- and *F*-dependent height and profile evolutions of the blazed nanograting.

### Time-dependent polymer reflow dynamics: evolution of slanted, parallelogrammatic nanograting

**Fig. 4:**
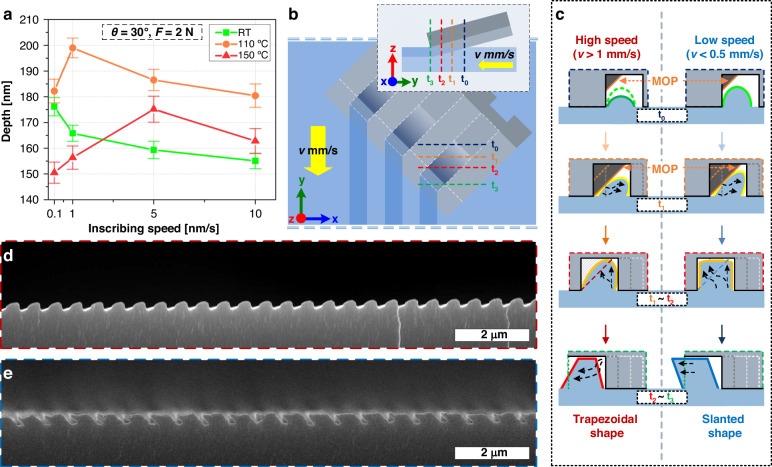
Time-dependent evolution of asymmetric nanogratings with diverse geometrical profiles, such as trapezoids and parallelograms. **a** Depths of nanogratings produced by ARC-DNI under parametrically controlled *v* values at various *T* values (*θ* and *F* are set to 30° and 2 N, respectively, in all cases). **b** Schematic top-view and side-view drawings of ARC-DNI performed at a non-zero *θ*. **c** Sequential diagrams of time-wise shape deformation of the polymer processed using ARC-DNI for comparing the high- (left side) and low (right side)-*v* cases. Cross-sectional SEM images of the nanogratings fabricated using the ARC-DNI process at **d** *v* = 1 mm/s and **e** v = 0.5 mm/s (*θ*, *T*, and *F* are set to 30°, 110 °C, and 3 N, respectively, in both cases)

Although the inscribing speed (*v*) has been fixed (to 1 mm/s) thus far, *v* has a significant influence on the depth and shape of the nanograting produced by ARC-DNI because it controls the time-dependent heat transfer and consequent dynamic flow behavior of the polymer^42,43^. Figure 4a shows plots of the nanograting depths obtained from the 30° azimuthally rotated, 2 N-applied ARC-DNI process executed with controlled ranges of *v* and *T* values. At 25 °C, the nanograting depth increases gradually as *v* decreases. At 25 °C, *F* is the only force driving plastic deformation (followed by elastic recovery) of the polymer substrate without any assistance provided by thermal reflow. Thus, as the contact time between the mold and the polymer substrate increases, the penetration depth of the mold increases when the substrate is moving. This indicates that generally, a longer inscribing time is required to achieve reliable polymer deformation under the mechanical force of the nanograting mold at 25 °C, for which a slower *v* in the ARC-DNI process is a favorable condition. However, the situation changes as *T* increases. The relaxation time, which represents the time required for structural recovery of the viscoelastic material, increases exponentially as the material temperature decreases from its *T*_*g*_^35,44,45^. As *T* approaches *T*_*g*_, thermal reflow, which generally tends to reduce the nanograting depth achieved by ARC-DNI, becomes more active, as previously depicted in Fig. 3b. In this scenario, an overly slow *v* can provide an excessive amount of thermal energy to the substrate owing to an increase in the heat transfer time, thereby decreasing the nanograting depth achieved by ARC-DNI. Accordingly, the *v* value required for creating the deepest nanograting varies with *T* as 0.1 mm/s, 1 mm/s, and 5 mm/s for 25 °C, 110 °C, and 150 °C, respectively (Fig. 4a). Specifically, the final nanograting depth that can be achieved by ARC-DNI can be determined using the tradeoff relationship between *v* and *T*; according to this relationship, the as-deformed polymer’s structural relaxation and thermal reflow compete with each other.

In ARC-DNI, the inscribing speed has a more pronounced effect on the pattern shape—beyond the pattern depth. By modulating *v* alone and keeping *θ* and *T* unchanged, the nanograting profile can be tuned from an asymmetric trapezoid to a slanted parallelogrammatic shape. Figure 4b–e collectively demonstrate this interesting finding. With a normal, relatively fast *v* (*i.e*., 1 mm/s; denoted as *v*_fast_), the nanograting structure achieved by ARC-DNI with the parameters *θ* = 30°, *T* = 110 °C, and *F* = 3 N has an asymmetric profile inclined toward the right (Fig. 4d). However, when *v* is reduced to 0.5 mm/s (denoted as *v*_slow_) without changing any other parameter, a parallelogram-profiled nanograting inclined toward the left is obtained (Fig. 4e). This remarkable *v*-dependent change in the resultant nanograting shape can be interpreted by considering two aspects: (1) the *v*-dependent heat transfer time that controls the local polymer viscosity and (2) the *v*-dependent “sidewall open time” (SOT; see definition below) that controls the directional reflow time. The difference in contact times between the left sidewall and the right sidewall (*t*_diff_) (defined as SOT) can be expressed as follows:

2 $${t}_{{diff}}={\lambda }_{{mold}}\times \sin (\theta )/v$$

where *v* is the inscribing speed of the ARC-DNI process.

Figure 4c presents a qualitative comparison between two scenarios: *v*_fast_ and *v*_slow_. When *v* is slower, heat transfer from the heated mold to the polymer can occur for a longer time, which decreases the polymer viscosity along the mold–substrate contact locus. This, in turn, increases the polymer’s thermal reflow per unit of time. By denoting the thermal reflows of the polymer over time for *v*_fast_ and *v*_slow_ as *q*_fast_ and *q*_slow_, respectively, the relationship between these values can be expressed as follows:

3 $${q}_{{fast}} \,<\, {q}_{{slow}}$$

Hence, the polymer is inscribed to a greater depth at a slower *v* in the initial period when the MOP does not yet affect the profile of the as-deformed polymer (at around *t*_*0*_). However, when the deformed polymer touches the MOP line along the lower-right direction, the polymer is forcibly deformed along the upper-right direction, and this process is maximized at *t*_*1*_. After *t*_*1*_, the MOP line retracts to open more space which the polymer “refills” between *t*_*1*_ and *t*_*2*_ (i.e., *t*_*2*_ – *t*_*1*_; defined as refilling time). This refilling time can be extended with a slower *v*; by denoting the refilling times in the *v*_*fast*_ and *v*_*slow*_ cases as *t*_*fast*_ and *t*_*slow*_, respectively, their values can be related as

4 $${t}_{{fast}} \,<\, {t}_{{slow}}$$

The total amount of refilled polymer can be expressed as *q* × *t* in both cases and according to Eq. (3) and (4), the following relationship holds:

5 $${q}_{{fast}}\times {t}_{{fast}}({\rm{for}}\,{v}_{{fast}}) \,<\, {q}_{{slow}}\times {t}_{{slow}}({\rm{for}}\,{v}_{{slow}})$$

which demonstrates that a larger quantity of polymer is refilled into the MOP area in a finite time when *v* is slower.

At this point, we consider the time required for the deformed polymer to be “slanted”. Following up on the discussion presented alongside Fig. 2, ARC-DNI uniquely induces a unidirectional reflow when the *t*_*diff*_ is generated. This factor is a representative feature of ARC-DNI because this occurs only if the *θ* exists. In detail, the left sidewall is the first to be released from contact (at time *t*_*2*_, as indicated in Fig. 4b), followed by the right sidewall (at *t*_*3*_). Specifically, the sidewall opens only toward the left (Fig. 4c) during the time *t*_*3*_ – *t*_*2*_ = *t*_*diff*_ (defined earlier as SOT). Consequently, the as-deformed polymer reflows unidirectionally during the SOT, leading to the formation of asymmetric nanogratings.

These aspects indicate that a slower *v* can allow for the directional reflow of a larger amount of polymer during a longer *t*_*diff*_, which helps to create a more slanted nanograting. Considering that the right sidewall continues to move toward the left as ARC-DNI proceeds, we can additionally infer that the nanograting structure extruded under the guidance of this “moving” boundary for a sufficiently long time can be shaped into a slanted nanograting having a parallelogrammatic profile inclined toward the left (Fig. 4e). While recapped here through a simplified comparison, finer control over *θ*, *T*, *F*, and *v* can be implemented for producing diverse application-specific asymmetric nanograting structures.

### Extending applicability: ARC-DNI on diverse materials and along diverse routes

**Fig. 5:**
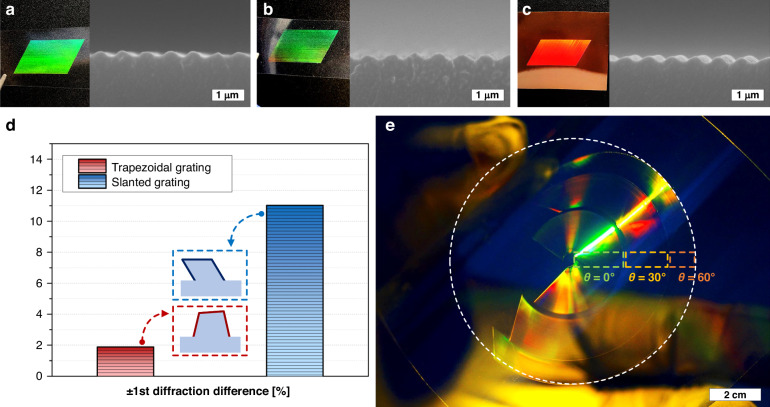
**Applicability of ARC-DNI on various substrates and along various routes**. **a** PET, **b** PFA, and **c** PI. **d** Difference in the measured first-order reflective diffraction efficiencies of asymmetric nanogratings with trapezoidal and slanted profiles produced by ARC-DNI on PC (schematically illustrated alongside). **e** Optical image of the lens-shaped diffractive gratings consisting of three concentric circular nanogratings fabricated by ARC-DNI along circular routes at *θ* values of 0°, 30°, and 60° (as marked in the image)

Meditating on the ARC-DNI principle, that is, mechanical-deformation-based patterning driven by a continuous inscription stroke, we can exploit several more practical perspectives. First, diverse materials softer than the mold can be readily patterned into the period- and shape-tunable nanogratings. Second, asymmetric nanogratings can be created by performing the inscription with an asymmetrically profiled mold instead of controlling *θ*. Third, period- and shape-controlled asymmetric nanogratings sweeping along specifically designed routes (*e.g*., free-curved, circular, or concentric nanograting) can be produced facilely by “steering” the inscription stroke. Fourth, the total processable area that can be covered with ARC-DNI is ~100 times larger than that covered by the original wafer mold because ARC-DNI only uses a sliced mold edge, as previously described^35^. Figure 5a–c show exemplary SEM images of the asymmetric nanogratings fabricated by executing 30°-rotated ARC-DNI (with *F* and *v* set to 1 mm/s and ~2–3 N, respectively) on various substrate materials other than PC, namely PET, PFA, and PI. It is noteworthy that, in each case, *T* was controlled to be slightly lower than the *T*_*g*_ of each film material to ensure reliable patterning: 75 °C for PET, 95 °C for PFA, and 325 °C for PI. A mold with an asymmetric, non-rectangular grating edge can be prepared, for instance, by fabricating the original grating structure on a (111)-plane Si wafer and cleaving it along the [01$\bar{1}$] or [10$\bar{1}$] orientation (*i.e*., tilted by ~30° with respect to the grating line)^46^. By using this mold to provide a triangular MOP and executing ARC-DNI without azimuthal angle modulation, continuous fabrication of asymmetric nanogratings can be realized. The experimental result is depicted in Fig. S6, which presents an asymmetric nanograting structure similar to that producible by 30°-rotated ARC-DNI by using a normal, rectangular grating mold.

The asymmetric trapezoidal and slanted nanograting structures that can be produced by controlled ARC-DNI can lead to intriguing asymmetric diffraction efficiencies, which are promising for the development of various DOEs, including high-efficiency optical waveguides and diffractive lenses^47–51^. As a simple demonstration, Fig. 5d shows the difference of measured first-order reflective diffraction efficiencies of the 50 nm-thick Ag-deposited trapezoidal and slanted nanogratings fabricated by performing ARC-DNI on the PC films with the same conditions used for fabricating the samples shown in Fig. 4d, e. While the total first-order diffraction efficiency of the trapezoidal grating is higher than that of the slanted grating due to a larger height of the trapezoidal grating (Table. S1), the difference between the first-order and negative first-order diffraction efficiencies is more distinct in the case of the slanted grating. This suggests that slanted nanogratings obtainable from an optimized ARC-DNI can be practically used for asymmetric waveguiding devices, which is indeed pursued as one of our successive works. Furthermore, ARC-DNI enables facile and continuous fabrication of circularly routed (simply “circular”) nanograting structures with controlled periods and shapes, which is promising from the perspective of realizing scalable diffractive lenses. While circular nanogratings are difficult to fabricate using conventional nanofabrication techniques, they can be fabricated swiftly by executing ARC-DNI along a circular route and properly maneuvering either the tool or substrate. Figure 5e shows an optical image of a lensed nanograting array consisting of three concentric nanogratings having various periods and profiles. They were fabricated by means of ARC-DNI at three different *θ* values of 0°, 30°, and 60° on a PC substrate rotated by three different radii of rotation. The diffraction color and brightness changed depending on *θ* (i.e., from the center of the circle toward the periphery), indicating that the diffraction angle of light varied according to the different periods and shapes of the nanogratings at each location. Currently, we are working to realize single-stroke fabrication of a diffractive lens with a scale of a few millimeters to tens of centimeters in order to minimize the collimation error that can occur because of the misalignment across multiple strokes.

By employing elaborate designs and fabrication conditions, a diffractive Fresnel lens and a thin film-type beam collimator, among others, can be fabricated using ARC-DNI as examples of practical photonic elements and high-resolution imaging systems^52–55^, respectively. This endeavor is in progress as a follow-up study. Lastly, we envision that ARC-DNI based on the continuous and scalable direct mechanical machining principle is well suited for “roll-to-roll (R2R)” patterning processes (e.g., R2R nanoimprint lithography (NIL) and rollable photolithography)^56–60^ and for other continuous mechanical nanopatterning techniques (e.g., periodic vibrational indentation)^61–63^ from the perspectives of extending productivity and applicability. Indeed, we are currently incubating several exciting ideas, such as churning out asymmetric nanogratings by performing R2R NIL using a flexible mold produced by ARC-DNI and manufacturing multiscale, multidimensional patterns by means of lithography or indenting specific micro- and nanoscale patterns over or beneath a nanograting produced by ARC-DNI.

## Conclusions

In summary, we developed the ARC-DNI process for continuous and scalable fabrication of asymmetric nanogratings with tunable periods, depths, and profiles. In ARC-DNI, a nanopatterned mold tool edge slides over a polymeric substrate at a controlled azimuthal angle to linearly inscribe an asymmetric grating pattern onto the substrate surface. By systematically investigating the important parameters, including the modulation of the tool stroke’s azimuthal angle, as well as the inscribing temperature, force, and speed, we demonstrated that asymmetric nanograting structures with various profiles, such as trapezoidal, blazed, and slanted profiles can be fabricated readily. The continuous machining principle of ARC-DNI, while requiring the extremely small area of a mold edge for large-area patterning, facilitates the practical manufacturing of asymmetric nanogratings on diverse polymeric materials and along diverse routes. This simple but powerful mechanism of ARC-DNI has an advantage for scalability compared to other lithographic techniques in terms of processing steps and system requirements, as discussed in Table. S2. While a lensed concentric asymmetric nanograting array was considered herein as an example, ARC-DNI can be utilized in a broader range of applications, including, but not limited to, optical and photonic elements, display and imaging system components, and sensors with large surface areas.

## Experimental and Methods

### Materials

The ARC-DNI tool (mold) used in this study was fabricated by cleaving the (100)-plane of a Si wafer containing a rectangular nanograting pattern with a period, depth, and duty ratio (i.e., a hill-to-valley ratio of grating) of 700 nm, 400 nm, and 1:1, respectively, along the direction perpendicular to the grating line. The detailed fabrication procedure of the Si nanograting structure can be found in the literature^64^. The width of the cleaved edge and the length of the mold tool were typically 20 mm and ~10 mm, respectively. The polymeric substrate films used in this study were composed of polycarbonate (PC) (DE-1-1, CLEAR, Makropol), polyethylene terephthalate (PET) (RX000, X type, Hyosung, Korea), perfluoroalkoxy (PFA) (PFA0125, Alphaflon, Korea), and polyimide (PI) (HN 1 mill, Kapton). These films were cut to proper sizes and cleaned with isopropyl alcohol, deionized water, and nitrogen blowing before use.

### ARC-DNI procedure

The ARC-DNI processing system was designed and built such that we could control the major process parameters, which, in this study, were the azimuthal angle (*θ*), mold temperature (*T*), the normal force (*F*), and substrate moving speed (*v*), by operating five-degrees-of-freedom (5-DOF) precision motion control stages (including DTG 60-200 (dovetail), DL 80-200 (*x*-*y*-axis), and DLJ 60-100 (lab-jack; *z*-axis) stages; all from DoDream System, Inc., Korea), a servo-motorized linear stage (LX1502-B1-N150, MISUMI), and digitally controlled and monitored microheater and load cells (see below for information pertaining to these instruments). The computer-aided design (CAD) assembly design and a completed prototype of the ARC-DNI system are illustrated in Fig. S2. The ARC-DNI procedure was as follows. First, a prepared mold was attached to the end of the mold arm by using a piece of double-sided Kapton tape (DHST, Korea). The mold arm was then mounted onto the system and the azimuthal angle (*θ*) was set to 0°, 30°, or 60° with the inscribing angle typically set to 35° (*i.e*., the interplanar angle between the mold and substrate planes, controlled by rotating the mold arm along the *y*-axis, as illustrated in Figs. S1 and S2). When *θ* was over 60°, MOP was too small to sustain inscribed nanogratings, thus the range of *θ* was set to be under 60°. Notably, while the inscribing angle and the depth and duty of the original mold were fixed in this study, they can be modulated to tune the MOP, as is being done in a follow-up study. The mold temperature (25–325 °C) was set using the stick-type microheater employed herein, a temperature sensor embedded in the mold arm (Hyundai Heating, Korea), and a temperature controller (TZ4M, Autonics Corp., Korea). The substrate was placed on the rubber pad attached to the top surface of the linear stage for moving the substrate. The rubber pad helped to prevent substrate slippage, reduce mold wear, and ensure uniform conformal contact between the mold edge and substrate surface. After additional 5-DOF alignment through fine adjustment of the motion stages, the mold edge was brought into contact with the substrate by using a controlled force (2 ~ 17 N) monitored by load cells (CB1A-K3, DACELL, Korea) connected to both ends of the stage through a digital indicator (DN-10W, DACELL, Korea). With the contact between the substrate and the mold at the set values of angle, temperature, and force, the substrate was conveyed by operating a servo motor (HF-KP053, Mitsubishi) at a controlled speed (0.1–10 mm/s). For the practical fabrication of optical devices, the ARC-DNI processing parameters, in this case, temperature, force, and inscribing speed, were set to the values that can achieve patterning depths over 80 nm. Specifically, the processing temperature was controlled from 25 °C to 150 °C, the inscribing force was applied from 2 N to 17 N, and the inscribing speed was set to 0.1–10 mm/s. To fabricate trapezoidal and slanted nanogratings for comparing reflective diffraction efficiencies, ARC-DNI was conducted on the PC films at the same conditions (*θ* = 30°, *F* = 2 N, *T* = 110 °C) except *v* (1 mm/s for trapezoidal gratings and 0.5 mm/s for slanted gratings). After that, a 50-nm thick Ag layer was deposited on the ARC-DNI-ed surfaces by an electron beam evaporation system (KVE-ENS4004, Korea Vacuum Tech, Korea) to increase reflective light intensity.

### Characterization

All scanning electron microscopy (SEM) images were obtained using a field emission scanning electron microscope (FE-SEM, JSM-6700F, JEOL Ltd.) operated at the typical voltage of 10 kV after sputtering a 2–3-nm-thick Pt film. For cross-sectional SEM imaging, the patterned polymer film (which was hardly cleavable because of its flexibility) was initially transferred onto polydimethylsiloxane (PDMS; Sylgard 184, Dow Corning Corp., with uncured PDMS poured on top of the film followed by curing at 40 °C for 5 h). The patterned PDMS was then transferred to an adhesion promoter (GAP-9200, MCNet Co., Ltd., Korea)-coated Si piece by using an ultraviolet (UV)-curable resin (PUM-3300, MCNet; cured for 1 min under 30 W using UV light with a peak wavelength of 365 nm). The resin-pattern-printed Si piece was cleaved to expose the nanopattern section. Quantitative data of the nanograting structures were obtained by analyzing the corresponding SEM images with image processing software (Java ImageJ, open source). An optical instrument setup for measuring reflective diffractive efficiency was described in Fig. S7. Here, a 532-nm diode-pumped solid-state laser (Thorlabs) was irradiated on a linear polarizer (Ø1/2” unmounted linear polarizers, Thorlabs) to form linearly polarized light. A pinhole with a diameter of 500 µm (P500HD-Ø1/2” (12.7 mm) mounted pinhole, Thorlabs) was used to block any unnecessary light. The intensity of light was measured using a photodiode power sensor (S120C, Thorlabs) equipped with a compact power and energy meter console (PM100D, Thorlabs). Finally, the reflective diffraction efficiency was calculated by dividing the measured intensity of the first-order diffracted light by the original intensity of the incident light. All data points presented with error bars in the plots denote the averaged values after repeating the measurements three to five times to ensure reliability and accuracy.

### Supplementary information

Supplemental material

## References

[1] Zeitner UD (2012). High performance diffraction gratings made by e-beam lithography. Appl. Phys. A.

[2] Zhou Y (2017). An electromagnetic scanning mirror integrated with blazed grating and angle sensor for a near infrared micro spectrometer. J. Micromech. Microeng..

[3] Sandfuchs O, Kraus M, Brunner R (2020). Structured metal double-blazed dispersion grating for broadband spectral efficiency achromatization. J. Opt. Soc. Am. A.

[4] Matricardi C (2020). High-Throughput Nanofabrication of Metasurfaces with Polarization-Dependent Response. Adv. Opt. Mater..

[5] Zhang Y, Fang F (2019). Development of planar diffractive waveguides in optical see-through head-mounted displays. Precis. Eng..

[6] Mattelin MA (2020). Design and fabrication of blazed gratings for a waveguide-type head mounted display. Opt. Express.

[7] Jeong Y (2021). Robust nanotransfer printing by imidization-induced interlocking. Appl. Surf. Sci..

[8] Gu T (2022). Low-threshold lasing behavior based on quasi-bound states in the continuum in a slanted guided-mode resonance nanocavity. Opt. Express.

[9] O’Shea, D. C., Suleski, T. J., Kathman, A. D. & Prather, D. W. Diffractive Optics: Design, Fabrication, and Test. SPIE Press: Bellingham; pp. 1–15, (2004).

[10] Nys I (2023). Geometric Phase Flat Optical Gratings with High Diffraction Angle Based on Dual-Frequency Nematic Liquid Crystal. Adv. Opt. Mater..

[11] Albero J (2013). Generalized diffractive optical elements with asymmetric harmonic response and phase control. Appl. Opt..

[12] Chen J (2018). Reducing electric-field-enhancement in metal-dielectric grating by designing grating with asymmetric ridge. Sci. Rep..

[13] Kress BC, Chatterjee I (2021). Waveguide combiners for mixed reality headsets: a nanophotonics design perspective. Nanophotonics.

[14] Bradley RM, Harper JME (1988). Theory of ripple topography induced By ION-bombardment. J. Vac. Sci. Technol. A.

[15] Bai B, Laukkanen J, Kuittinen M, Siitonen S (2010). Optimization of nonbinary slanted surface-relief gratings as high-efficiency broadband couplers for light guides. Appl. Opt..

[16] Voronov DL (2011). Fabrication and characterization of ultra-high resolution multilayer-coated blazed gratings. Nucl. Instrum. Methods Phys. Res. Sect. A.

[17] Jeong HE (2009). A nontransferring dry adhesive with hierarchical polymer nanohairs. Proc. Natl Acad. Sci. USA..

[18] Gritsai Y, Goldenberg LM, Stumpe J (2011). Efficient single-beam light manipulation of 3D microstructures in azobenzene-containing materials. Opt. Express.

[19] Ok JG, Ahn SH, Kwak MK, Guo LJ (2013). Continuous and high-throughput nanopatterning methodologies based on mechanical deformation. J. Mater. Chem. C..

[20] Ok JG, Panday A, Lee T, Jay Guo L (2014). Continuous fabrication of scalable 2-dimensional (2D) micro- and nanostructures by sequential 1D mechanical patterning processes. Nanoscale.

[21] Ok JG, Shin YJ, Park HJ, Guo LJ (2015). A step toward next-generation nanoimprint lithography: extending productivity and applicability. Appl. Phys. A.

[22] Mi XT (2019). Ruling engine using adjustable diamond and interferometric control for high-quality gratings and large echelles. Opt. Express.

[23] Shen C (2018). Convex blazed grating of high diffraction efficiency fabricated by swing ion-beam etching method. Opt. Express.

[24] Roeder M (2019). Fabrication of curved diffractive optical elements by means of laser direct writing, electroplating, and injection compression molding. J. Manuf. Process..

[25] Xu S (2020). High-Efficiency Fabrication of Geometric Phase Elements by Femtosecond-Laser Direct Writing. Nanomaterials.

[26] Del Campo A, Arzt E (2008). Fabrication approaches for generating complex micro- and nanopatterns on polymeric surfaces. Chem. Rev..

[27] Huang WB (2017). A review of the scalable nano-manufacturing technology for flexible devices. Front. Mech. Eng..

[28] Gao J (2021). A review on fabrication of blazed gratings. J. Phys. D..

[29] Huang YJ, Chang TL, Chou HP, Lin CH (2008). A novel fabrication method for forming inclined groove-based microstructures using optical elements. Jpn. J. Appl. Phys..

[30] Mekaru H, Koizumi O, Ueno A, Takahashi M (2010). Inclination of mold pattern’s sidewalls by combined technique with photolithography at defocus-positions and electroforming. Microsyst. Technol..

[31] Jirigalantu (2016). Ruling of echelles and gratings with a diamond tool by the torque equilibrium method. Appl. Opt..

[32] Berdin, A., Rekola, H. T. & Priimagi, A. Complex Fourier Surfaces by Superposition of Multiple Gratings on Azobenzene Thin Films. *Adv. Opt. Mater*. 2301597 (2023).

[33] Ding P (2023). Fabrication of Optical Fourier Surface by Multiple-Frequency Vibration Cutting for Structural True Coloration. Small.

[34] Oh DK (2019). Facile and Scalable Fabrication of Flexible Reattachable lonomer Nanopatterns by Continuous Multidimensional Nanoinscribing and Low-temperature Roll Imprinting. ACS Appl. Mater. Interfaces.

[35] Oh DK (2019). Tailored Nanopatterning by Controlled Continuous Nanoinscribing with Tunable Shape, Depth, and Dimension. ACS Nano.

[36] Chen L (2021). Size-Selective Sub-micrometer-Particle Confinement Utilizing Ionic Entropy-Directed Trapping in Inscribed Nanovoid Patterns. ACS Nano.

[37] Lee W (2021). Solution-processable electrode-material embedding in dynamically inscribed nanopatterns (SPEEDIN) for continuous fabrication of durable flexible devices. Microsyst. Nanoeng..

[38] Oh DK (2022). Burr- and etch-free direct machining of shape-controlled micro- and nanopatterns on polyimide films by continuous nanoinscribing for durable flexible devices. Microelectron. Eng..

[39] Kirchner R, Schleunitz A, Schift H (2014). Energy-based thermal reflow simulation for 3D polymer shape prediction using Surface Evolver. J. Micromech. Microeng..

[40] Kirchner R, Schift H (2019). Thermal reflow of polymers for innovative and smart 3D structures: A review. Mater. Sci. Semiconductor Process..

[41] Yang F (1997). Viscosity measurement of polycarbonate by using a penetration viscometer. Polym. Eng. Sci..

[42] Heyderman LJ (2000). Flow behaviour of thin polymer films used for hot embossing lithography. Microelectron. Eng..

[43] Rowland HD, King WP (2004). Polymer deformation and filling modes during microembossing. J. Micromech. Microeng..

[44] Shaw, M. T. & MacKnight, W. J. Introduction to Polymer Viscoelasticity. Wiley: Hoboken; pp. 107–128, (2005).

[45] Fan BF, Kazmer DO (2005). Low-temperature modeling of the time-temperature shift factor for polycarbonate. Adv. Polym. Technol..

[46] Lei W-S, Kumar A, Yalamanchili R (2012). Die singulation technologies for advanced packaging: A critical review. J. Vac. Sci. Technol. B.

[47] Bernard CK, Maria P (2022). Holographic optics in planar optical systems for next generation small form factor mixed reality headsets. Light-Adv. Manuf..

[48] Dewen C (2021). Design and manufacture AR head-mounted displays: A review and outlook. Light-Adv. Manuf..

[49] Levola T, Laakkonen P (2007). Replicated slanted gratings with a high refractive index material for in and outcoupling of light. Opt. Express.

[50] Bin W, Jianhua J, Nordin GP (2005). Embedded slanted grating for vertical coupling between fibers and silicon-on-insulator planar waveguides. IEEE Photonics Technol. Lett..

[51] Miller JM (1997). Design and fabrication of binary slanted surface-relief gratings for a planar optical interconnection. Appl. Opt..

[52] Siemion A (2019). Terahertz Diffractive Optics-Smart Control over Radiation. J. Infrared Millim. Terahertz Waves.

[53] Lin MY, Chuang CH, Chou TA, Chen CY (2021). A theoretical framework for general design of two-materials composed diffractive fresnel lens. Sci. Rep..

[54] Languy F (2011). Flat Fresnel doublets made of PMMA and PC: combining low cost production and very high concentration ratio for CPV. Opt. Express.

[55] Park J (2021). Demonstration of the one-step continuous fabrication of flexible polymer ridge waveguides via nanochannel-guided lithography. J. Ind. Eng. Chem..

[56] Ok JG (2012). Continuous and scalable fabrication of flexible metamaterial films via roll-to-roll nanoimprint process for broadband plasmonic infrared filters. Appl. Phys. Lett..

[57] Kwak MK, Ok JG, Lee JY, Guo LJ (2012). Continuous phase-shift lithography with a roll-type mask and application to transparent conductor fabrication. Nanotechnology.

[58] Ok JG (2013). Photo-roll lithography (PRL) for continuous and scalable patterning with application in flexible electronics. Adv. Mater..

[59] Wi JS (2017). Facile three-dimensional nanoarchitecturing of double-bent gold strips on roll-to-roll nanoimprinted transparent nanogratings for flexible and scalable plasmonic sensors. Nanoscale.

[60] Wi JS, Oh DK, Kwak MK, Ok JG (2018). Size-dependent detection sensitivity of spherical particles sitting on a double-bent gold strip array. Opt. Mater. Express.

[61] Lee S (2021). Piezo-Actuated One-Axis Vibrational Patterning for Mold-Free Continuous Fabrication of High-Precision Period-Programmable Micro- and Nanopatterns. ACS Nano.

[62] Ahn SH (2013). Template-Free Vibrational Indentation Patterning (VIP) of Micro/Nanometer-Scale Grating Structures with Real-Time Pitch and Angle Tunability. Adv. Funct. Mater..

[63] Oh DK (2021). Nanoimprint lithography for high-throughput fabrication of metasurfaces. Front. Optoelectron..

[64] Ok JG (2011). Continuous patterning of nanogratings by nanochannel-guided lithography on liquid resists. Adv. Mater..

