# Supplementary material for: Azimuthal rotation-controlled nanoinscribing for continuous patterning of period- and shape-tunable asymmetric nanogratings
Source: Microsyst Nanoeng. 2024 May 11;10:60. doi: 10.1038/s41378-024-00687-4 (PMC11088629; doi:10.1038/s41378-024-00687-4)
Supplement: Supplementary file 1 — Supplemental material [file 41378_2024_687_MOESM1_ESM.docx]

**Supplementary Information**

**Azimuthal rotation-controlled nanoinscribing for continuous patterning of period- and shape-tunable asymmetric nanogratings**

Useung Lee^1,†,‡^, Hyein Kim^1,†^, Dong Kyo Oh^2,†^, Nayeong Lee^1,†^, Jonggab Park^1^, Jaewon Park^1^, Hyunji Son^1^, Hyunchan Noh^1,§^, Junsuk Rho^2,3,4,5^, and Jong G. Ok^1,^*

^1^ Department of Mechanical and Automotive Engineering, Seoul National University of Science and Technology, 232 Gongneung-ro, Nowon-gu, Seoul 01811, Republic of Korea

^2^ Department of Mechanical Engineering, Pohang University of Science and Technology (POSTECH), Pohang 37673, Republic of Korea

^3^ Department of Chemical Engineering, Pohang University of Science and Technology (POSTECH), Pohang 37673, Republic of Korea

^4^ POSCO-POSTECH-RIST Convergence Research Center for Flat Optics and Metaphotonics, Pohang 37673, Republic of Korea

^5^ National Institute of Nanomaterials Technology (NINT), Pohang 37673, Republic of Korea

^‡^Current address: Department of Mechanical Engineering, Korea University, 145 Anam-ro, Seongbuk-gu, Seoul 02841, Republic of Korea

^§^Current address: Research Team, Hyundai Motor Group, 150 Hyundaiyeonguso-ro, Hwaseong-si, Gyeonggi 18280, Republic of Korea

^†^These authors contributed equally to this work.

*Corresponding author: E-mail: [jgok@seoultech.ac.kr](mailto:jgok@seoultech.ac.kr), Tel. +82-2-970-9012

**Supplementary Figures**


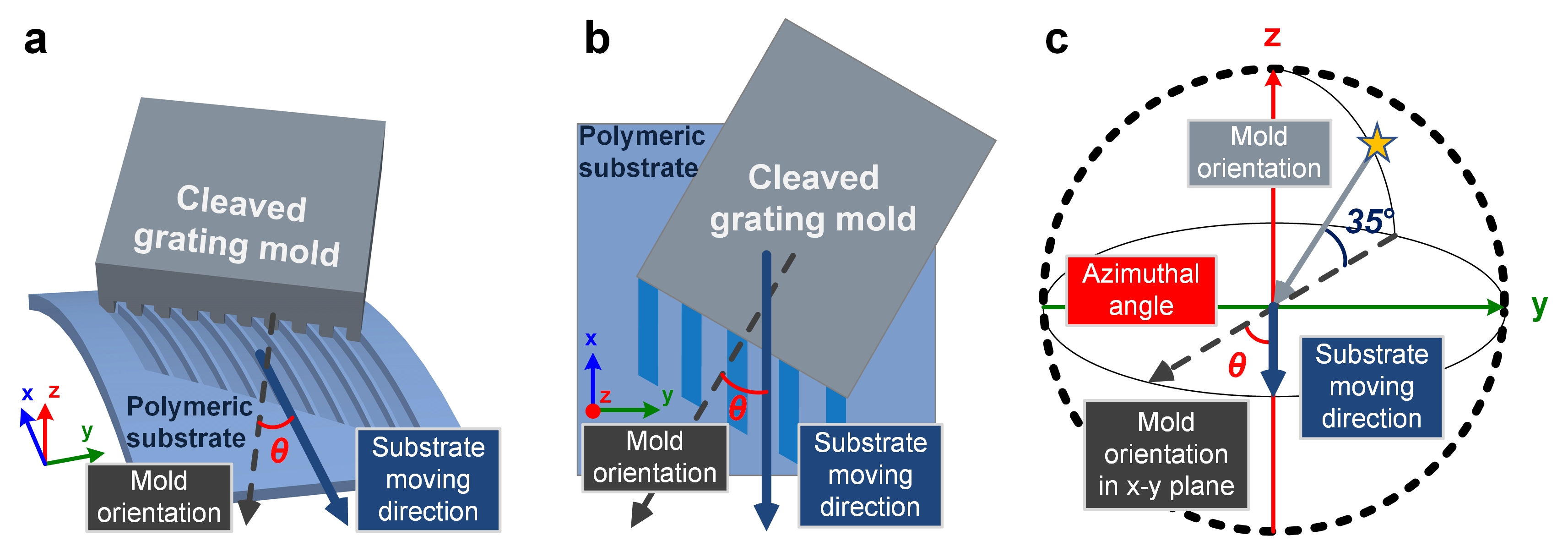


**Figure S1**. (a) Perspective 3D scheme and (b) normal 2D x-y plane view, describing that the azimuthal angle (*θ*) of the ARC-DNI system is defined as the angle between the master mold orientation and the substrate moving direction. (c) Simplified spherical coordinate system expressing azimuths and directions of the mold and the substrate.


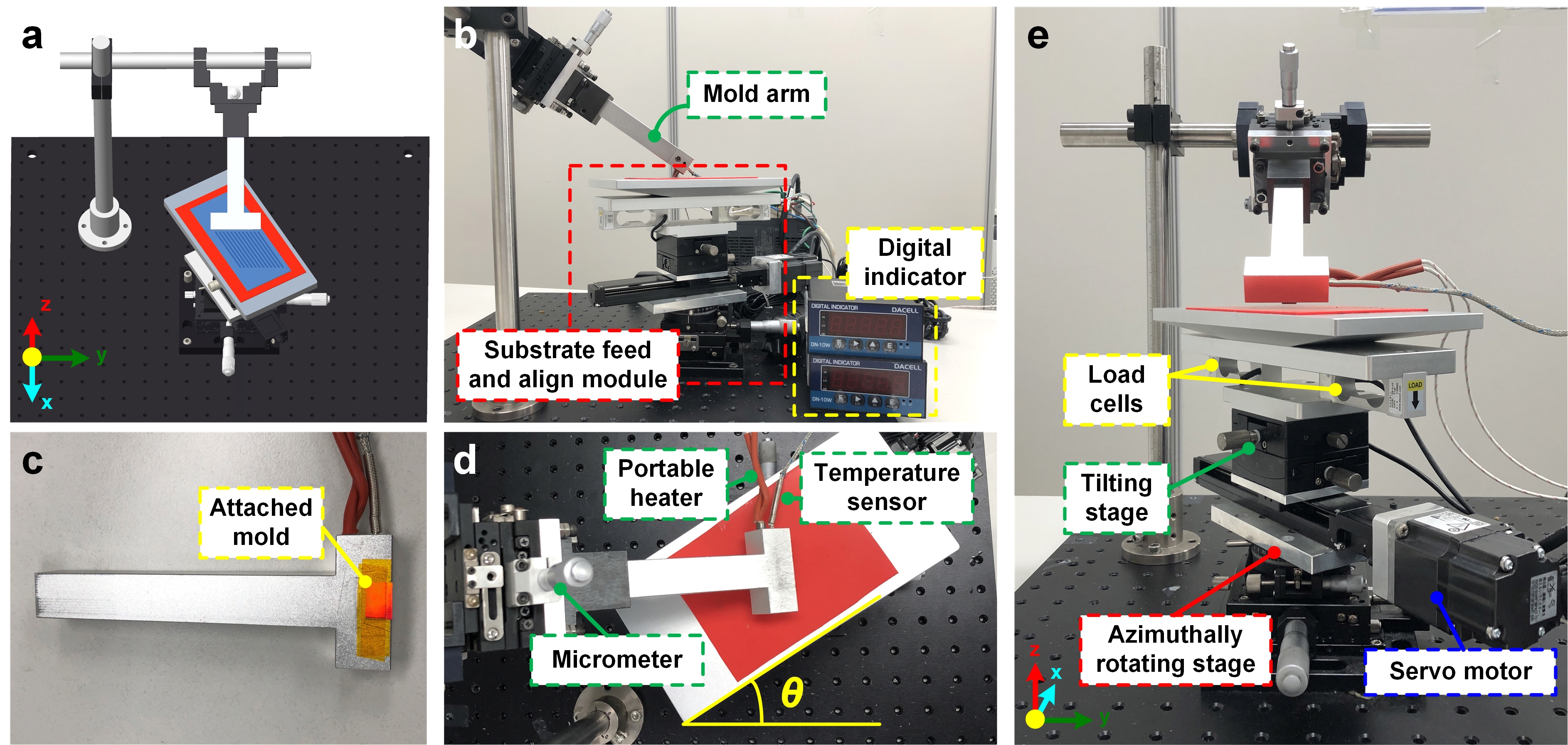


**Figure S2.** The ARC-DNI processing system: (a) 3D CAD design drawn by using a PTC Creo Parametric 6.0 software and (b-e) optical photos of the prototype. The system consists of three main modules: mold arm equipped with a microheater and a temperature sensor, precision motion control stage stack integrated with a couple of load cells, and digital temperature and force controllers, as shown in (b). As can be seen in (c), the mold arm has a head inside which a microheater stick and a temperature sensor are installed, where a cleaved nanograting mold can be attached. The azimuth (*θ*), defined in the main text is visually marked in (d). The load cells are installed underneath the substrate loading plate motorized by a servo motor, as shown in (e).


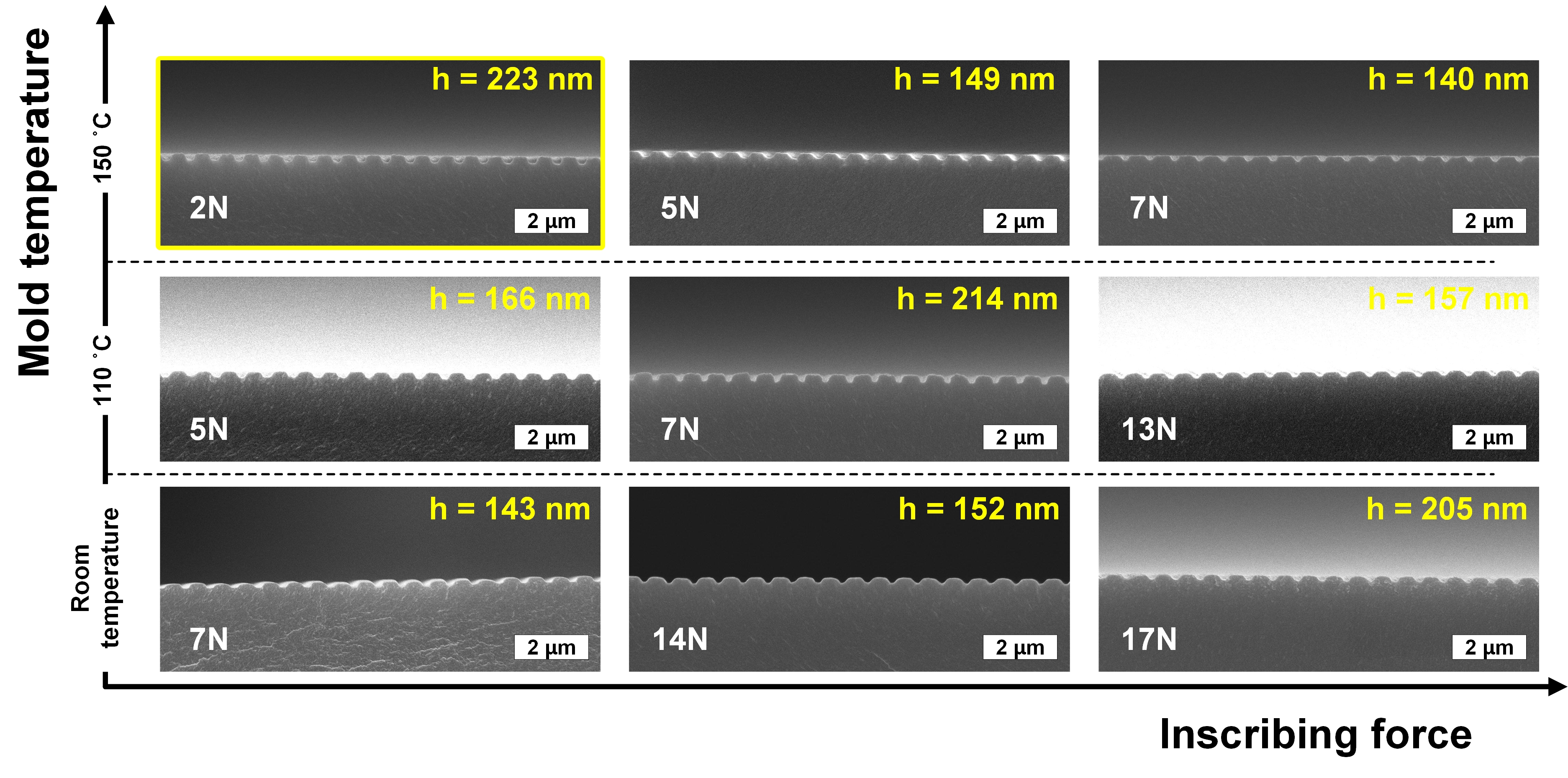


**Figure S3.** Cross-sectional SEM images of the nanogratings fabricated with the ARC-DNI process at ***θ* = 0°**, matricized by the mold temperature (*T*) and inscribing force (*F*) conditions (simply referred to as ‘*T*–*F* matrix’ hereafter). For all cases shown in **Figure S2** and in **Figures S3** and **S4** as well, the inscribing speed was set to 1 mm/s, and the yellow-boxed images indicate the *T*–*F* condition giving the deepest (highest) nanograting for the corresponding *θ*.


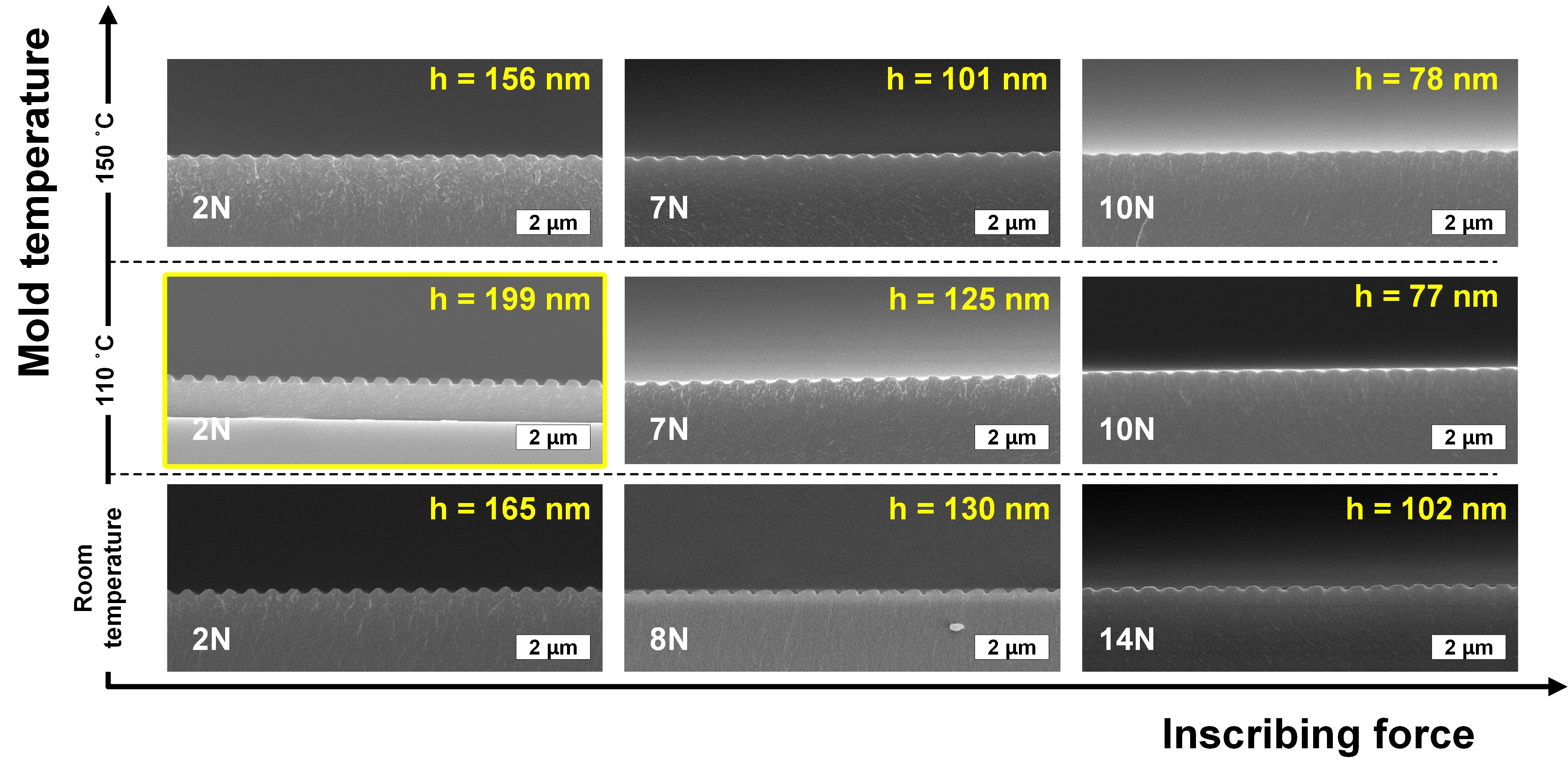


**Figure S4.** *T*–*F* matrix of the nanogratings fabricated with the ARC-DNI process at ***θ* = 30°**.


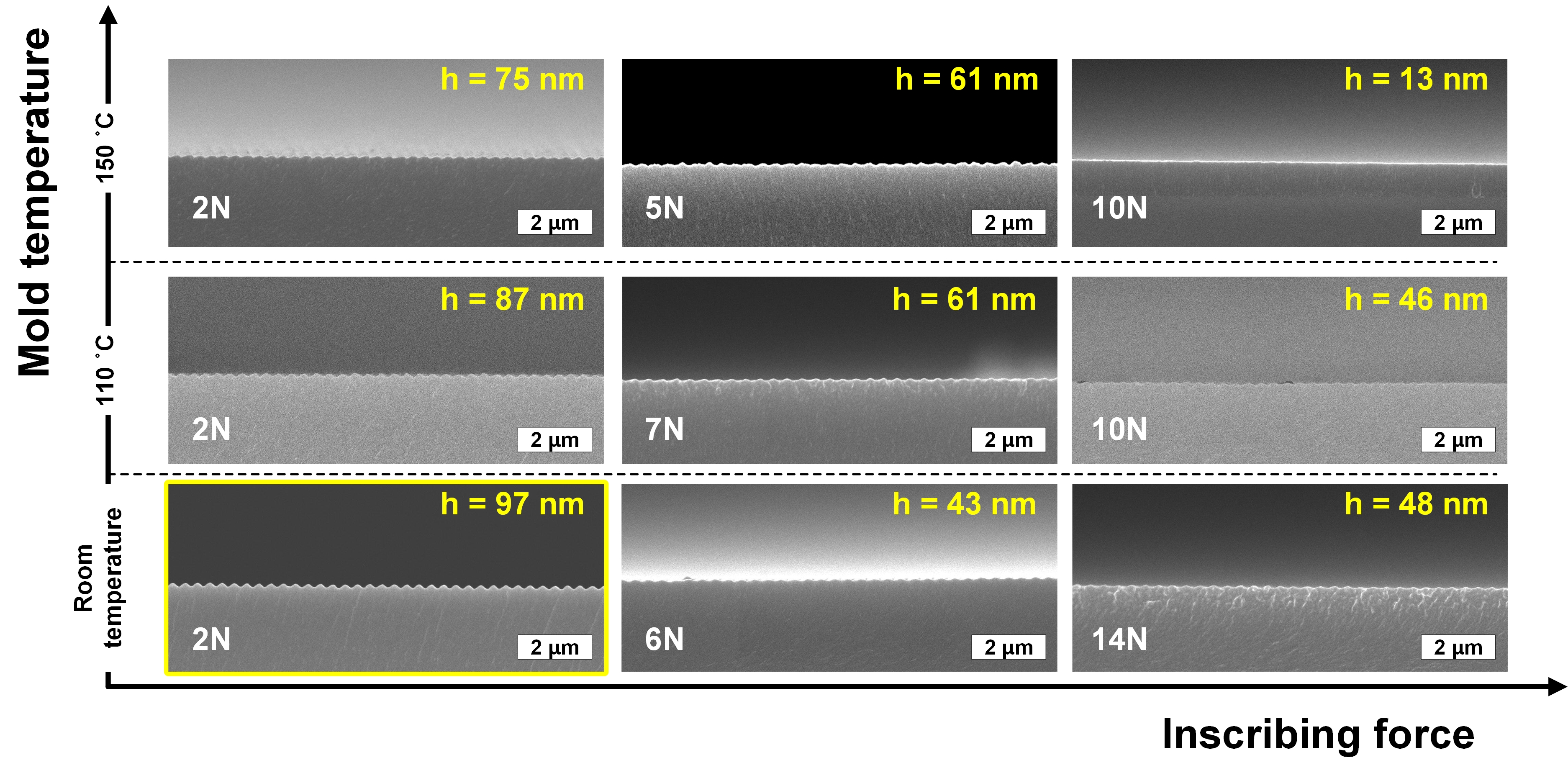


**Figure S5.** *T*–*F* matrix of the nanogratings fabricated with the ARC-DNI process at ***θ* = 60°**.


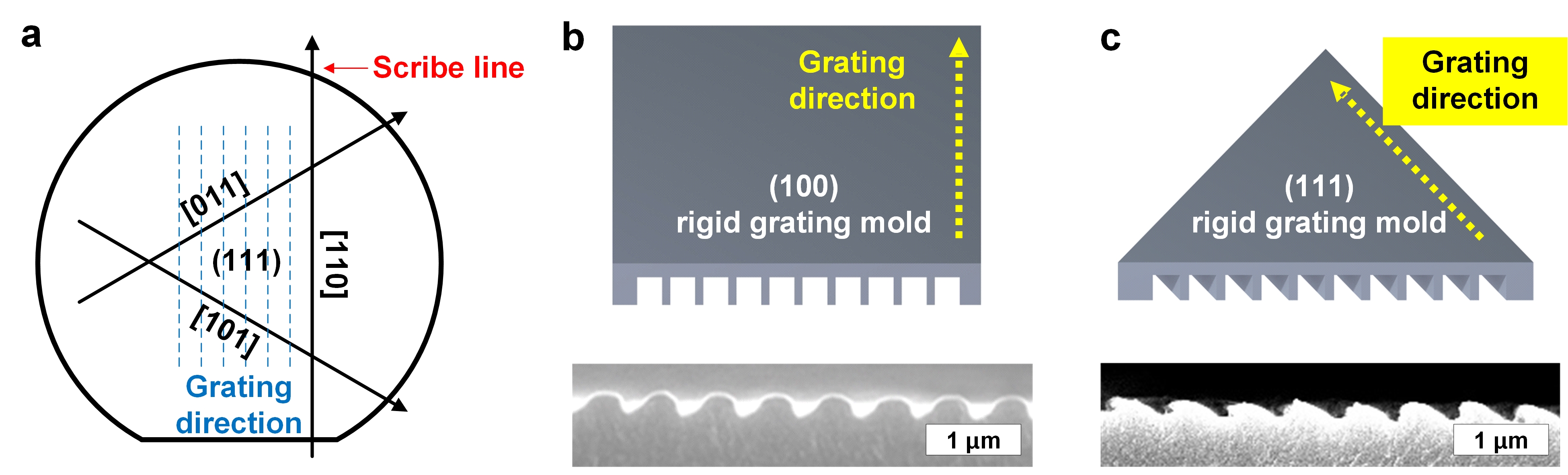


**Figure S6.** (a) Schematic drawing of Miller indices of crystal structures of a (111)-plane Si wafer. The dashed lines indicate the nanograting lines and the [011]/[101] lines show the typical scribe lines for cleaving. Schemes and representative SEM images of the nanograting molds and ARC-DNI results were obtained by using (b) (100)-plane Si and (c) (111)-plane Si wafers. *θ* in ARC-DNI was 30° and 0° for (b) and (c), respectively. For both cases, the following conditions were used: *T* = 110 °C, *F* = 2 N, and *v* = 1 mm/s.

**Table S1.** Measured 1^st^ diffraction efficiency of trapezoidal and slanted nanogratings.

|  | 1^st^ diffraction efficiency (%) | - 1^st^ diffraction efficiency (%) |
| --- | --- | --- |
| Trapezoidal nanograting | 25.749 | 27.633 |
| Slanted nanograting | 11.667 | 22.689 |


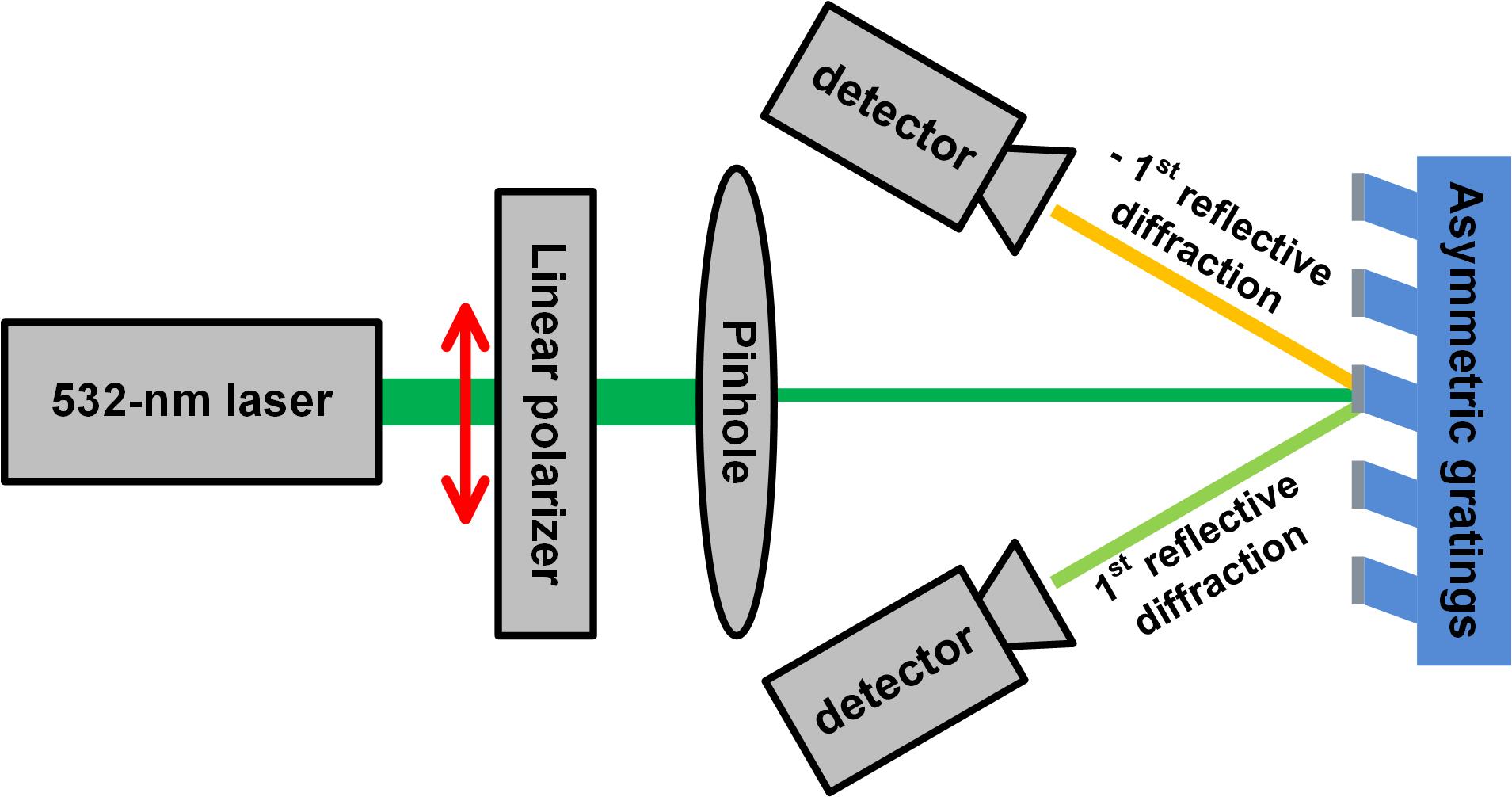


**Figure S7.** Schematic drawing of an optical instrument setup to measure first-order diffraction efficiencies in the far field.

**Table S2.** Comparison of lithographic processes and ARC-DNI to fabricate asymmetric or slanted nanogratings.

|  | Lithography  + etching | Nanoimprint lithography | ARC-DNI |
| --- | --- | --- | --- |
| Processing steps | > 6  (PR coating – lithography – develop – etch mask deposition – etching – etch mask removal) | 5  (mold prep. – surface treatment – resin coating – imprint – demolding) | 3  (mold prep. – pressure and heat – inscribing) |
| Difficulty of masks or molds | Easy to make masks (grating shape) | Difficult to make asymmetric molds  (will need lithography + etching process) | Easy to make molds (rectangular shape) |
| Need of vacuum process | Essential | Occasional | None |
| Cost | $$$$ | $$ | $ |
| Substrate | Flat & rigid | Flexible | Flexible |
| Resolution | Extremely high | High | Moderate |
| Dimension | 2D | 2D | 1D > 2D |
| Reference | [S1] | [S2],[S3] | This work |

**References**

S1. El-Refaei, H., Yevick, D. & Jones, T. Slanted-Rib Waveguide InGaAsP-InP Polarization Converters. *Journal of Lightwave Technology* **22**, 1352 (2004).

S2. Jeong, H. E. *et al.* A nontransferring dry adhesive with hierarchical polymer nanohairs. *Proc. Natl. Acad. Sci. U. S. A.* **106**, 5639 (2009).

S3. Levola, T. & Laakkonen, P. Replicated slanted gratings with a high refractive index material for in and outcoupling of light. *Opt. Express* **15**, 2067-2074 (2007).
